# The Homeobox Transcription Factor *Barx2* Regulates Plasticity of Young Primary Myofibers

**DOI:** 10.1371/journal.pone.0011612

**Published:** 2010-07-15

**Authors:** Robyn Meech, Mariana Gomez, Christopher Woolley, Marietta Barro, Julie-Ann Hulin, Elisabeth C. Walcott, Jary Delgado, Helen P. Makarenkova

**Affiliations:** 1 The Scripps Research Institute, La Jolla, California, United States of America; 2 The Flinders University of South Australia, Beford Park, South Australia, Australia; 3 The Neurosciences Institute, San Diego, California, United States of America; Childrens Hospital Los Angeles, United States of America

## Abstract

**Background:**

Adult mammalian muscle retains incredible plasticity. Muscle growth and repair involves the activation of undifferentiated myogenic precursors called satellite cells. In some circumstances, it has been proposed that existing myofibers may also cleave and produce a pool of proliferative cells that can re-differentiate into new fibers. Such myofiber dedifferentiation has been observed in the salamander blastema where it may occur in parallel with satellite cell activation. Moreover, ectopic expression of the homeodomain transcription factor *Msx1* in differentiated C2C12 myotubes has been shown to induce their dedifferentiation. While it remains unclear whether dedifferentiation and redifferentiaton occurs endogenously in mammalian muscle, there is considerable interest in induced dedifferentiation as a possible regenerative tool.

**Methodology/Principal Findings:**

We previously showed that the homeobox protein Barx2 promotes myoblast differentiation. Here we report that ectopic expression of Barx2 in young immature myotubes derived from cell lines and primary mouse myoblasts, caused cleavage of the syncytium and downregulation of differentiation markers. Microinjection of Barx2 cDNA into immature myotubes derived from primary cells led to cleavage and formation of mononucleated cells that were able to proliferate. However, injection of Barx2 cDNA into mature myotubes did not cause cleavage. Barx2 expression in C2C12 myotubes increased the expression of cyclin D1, which may promote cell cycle re-entry. We also observed differential muscle gene regulation by *Barx2* at early and late stages of muscle differentiation which may be due to differential recruitment of transcriptional activator or repressor complexes to muscle specific genes by *Barx2*.

**Conclusions/Significance:**

We show that *Barx2* regulates plasticity of immature myofibers and might act as a molecular switch controlling cell differentiation and proliferation.

## Introduction

Adult mammalian muscle has the potential to regenerate by activation of undifferentiated myogenic precursor cells (satellite cells), which are normally quiescent and situated between the basal membrane and the myofibers [1], [2], [3], [4]. Upon activation, satellite cells divide asymmetrically producing daughter cells with different fates [5], [6]. One daughter cell proceeds to proliferation and myogenic differentiation and the other may return to the quiescent satellite cell pool [7], [8]. Urodele amphibians show much greater regenerative plasticity than that of mammals, undergoing epimorphic regeneration in which whole structures, rather than isolated tissues are reformed [9], [10]. This ability has been described for many urodele organs, including lens, retina, intestine, tail and limbs [11], [12]. Previous experiments have shown that after amphibian limb amputation, stump tissues form a structure called the blastema in which cellular dedifferentiation occurs, producing a pool of progenitor-like cells that participate in regeneration [13], [14]. For example, dedifferentiation of damaged amphibian myofibers produces a pool of proliferating progenitor cells that can re-differentiate to form new muscle.

It was previously suggested that dedifferentiated muscle cells became multipotent and could contribute to development of not only new muscle but also cartilage and bones [15]; however, more recent work refutes this [16], [17]. Although all cells in the newly formed blastema have a very similar morphology, immunostaining with tissue-specific markers has revealed heterogeneity of the blastema cells [10], [18]. In particular, a very recent study showed that the blastema is a heterogeneous collection of progenitor cells with restricted fates [16]. These experiments indicate that dedifferentiating muscle remains restricted to the muscle lineage [16]. Moreover, recent work suggests that the regeneration of muscle during epimorphic limb regeneration involves not only dedifferentiation of damaged myofibers, but also the activation of muscle satellite cells as in mammals [12], [19]. These data suggest that amphibian and mammalian regeneration share more similarity than was originally apparent.

Factors controlling dedifferentiation in newt limb are not well understood; however, expression of the muscle segment homeobox gene *msx-1* is induced in the blastema [20] and also in regenerating amputated mammalian digits [21], suggesting a role in dedifferentiation. This transcription factor is also expressed in migrating limb muscle precursors preventing them from premature differentiation [22], [23], [24]. While mammalian myofibers appear much less plastic than those of the amphibian, it has been demonstrated that ectopic expression of *msx1* in mouse C2C12 myotubes induces cleavage of myotubes into proliferating, mononucleated cells [25]. Moreover these cells appear able to re-differentiate into new myofibers [25], [26]. This suggests that the molecular and cellular machinery that underpins functional dedifferentiation is present in mammalian muscle. There is currently no convincing evidence that dedifferentiation occurs naturally after injury of mammalian muscle in vivo, and if it occurs it is unlikely to be a major contributor to normal muscle regeneration. However, as recent work on induced pluripotent stem cells (iPS cells) has shown, even synthetic approaches to reprogramming differentiated cells can have important ramifications for basic biology and lead to new avenues in regenerative medicine [27], [28]. Thus defining the molecular mechanisms and factors involved in induced dedifferentiation in mammals may lead to development of new techniques for control of cell and tissue plasticity.

In this study we investigated the role of the homeodomain transcription factor *Barx2* in regulation of myofiber plasticity. During early embryonic stages of mouse development, *Barx2* is widely expressed in proliferating and differentiating cartilage and muscle tissue [29]. However, in adult mice *Barx2* expression was found to be restricted to the joint region [29] and also to muscle satellite cells [30]. We recently reported that *Barx2* cooperates with other muscle-expressed transcription factors to regulate cytoskeletal remodeling events of early myoblast differentiation [30].

Here we show that ectopic expression of Barx2 in C2C12 myotubes and MyoD-induced C3H10T1/2 myotubes induced apparent dedifferentiation indicated by myotube cleavage and concomitant down-regulation of muscle differentiation markers. We also extended these studies to differentiated primary mouse muscle cell cultures; we observed two types of myotubes in primary cultures: thin slowly contracting myotubes with nuclei aligned along the middle of the fiber (immature myotubes), and thicker, faster contracting myotubes often with small or large nuclei clusters (mature myotubes). Microinjection of Barx2 cDNA into the immature myotubes induced cleavage and formation of mononucleated cells that were able to proliferate, whereas injection of Barx2 cDNA into mature myotubes induced myotube contraction but not cleavage. Thus our data indicate that only immature myotubes can dedifferentiate in response to *Barx2* over-expression, while more mature myotubes appear to have lost this ability. These results also suggest that *Barx2*, like *Msx1*, can regulate muscle plasticity and that homeobox factors could be a part of a general mechanism that controls the susceptibility of cells to reprogramming.

## Results

**Figure 1:**
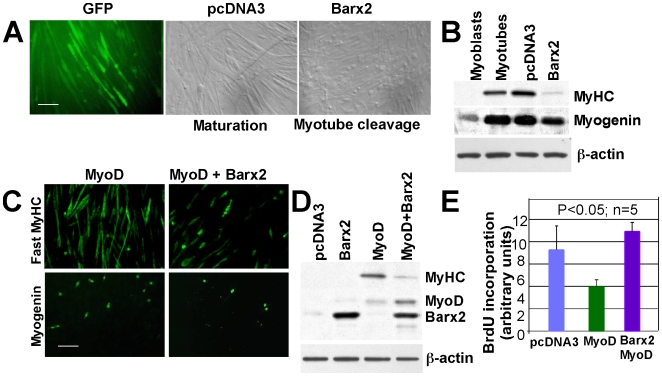
Ectopic expression of Barx2 in differentiated myotubes causes their dedifferentiation. **A.** Serum-deprived C2C12 cultures contaning many early myotubes were transfected with either Barx2/pcDNA, empty pcDNA3, or GFP-expressing plasmids. Images of maturing or apparently fragmenting myotubes were taken after 3–4 days, **B.** Levels of the differentiation-associated proteins myogenin and myosin heavy chain (MyHC) were measured by immunoblotting of total protein from Barx2-expressing and control cultures corresponding to those shown in A. Untransfected myoblast and myotube cultures were also analysed as a reference. **C.** C3H10T1/2 mesenchymal progenitor cells were transfected with either MyoD/pcDNA3 or a combination of MyoD/pcDNA3 and Barx2/pcDNA3 plasmids and differentiation was subsequently induced by serum withdrawal for 2–3 days. The proportion of cells expressing myogenin and MyHC and appearance of myotubes was assessed by immunostaining. **D.** MyHC protein was assessed by immunoblotting of total protein from cultures corresponding to those shown in **C** and **D.** Analysis of BrdU incorporation was performed in C3H10T1/2 cultures corresponding to those shown in **C**; co-expression of Barx2 with MyoD increased numbers of proliferating cells relative to MyoD alone - **E**.

We previously found that Barx2 is expressed in embryonic myoblasts and satellite cells *in vivo* and is upregulated early during differentiation of C2C12 and C3H10T1/2 cells and primary myoblasts in culture. However, we do not observe Barx2 in the nuclei of mature myofibres ([30], [31] and unpublished data). To examine the effects of ectopic Barx2 expression in differentiated muscle cells, a *Barx2* expression construct or control vector were transfected into serum-deprived C2C12 cultures that were comprised primarily of early-stage myotubes. Expression of GFP demonstrates the high efficiency of transfection (Fig. 1A). Within 3–4 days, Barx2-expressing cultures showed a remarkable reduction in myotube numbers due to apparent myotube fragmentation (Fig. 1A). In contrast, control pcDNA3-transfected cultures showed only maturing myotubes. Consistent with this result, cultures ectopically expressing Barx2 showed decreased levels of the differentiation-associated proteins myogenin and myosin heavy chain (MyHC) (Fig.1B).

To further explore these phenomena, we performed similar experiments with C3H10T1/2 mesenchymal progenitor cells that were induced to undergo terminal differentiation and form myotubes by transfection of MyoD and subsequent serum withdrawal (Fig. 1C). As with C2C12 cells, coexpression of Barx2 and MyoD in these cells reduced myogenin (Fig. 1C) and MyHC expression (Fig. 1C and 1D) and caused apparent cleavage of myotubes with many shorter myotubes and mononucleated cells present.

BrdU labeling showed the expected decrease in proliferating cells in MyoD-transfected C3H10T1/2 cultures relative to pcDNA3-transfected cells after several days in differentiation media. This decrease was completely blocked by co-expression of Barx2 (Fig.1E). This could indicate that *Barx2* impairs differentiation; however, our previous work has shown that *Barx2* in fact stimulates the earliest phases of differentiation in cooperation with *MyoD* [30]. Thus we suggest that this result, together with our observations of myotube fragmentation, further supports the idea that *Barx2* promotes dedifferentiation of terminally differentiated myotubes. However, a clear caveat of the proliferation analysis is that a subpopulation of mononucleated cells is always present in the myotube cultures and proliferating cells may derive from these cells rather than dedifferentiated myotubes. To specifically address this concern we performed further experiments with a single-cell-tracing technique and primary muscle cells as described below.

**Figure 2:**
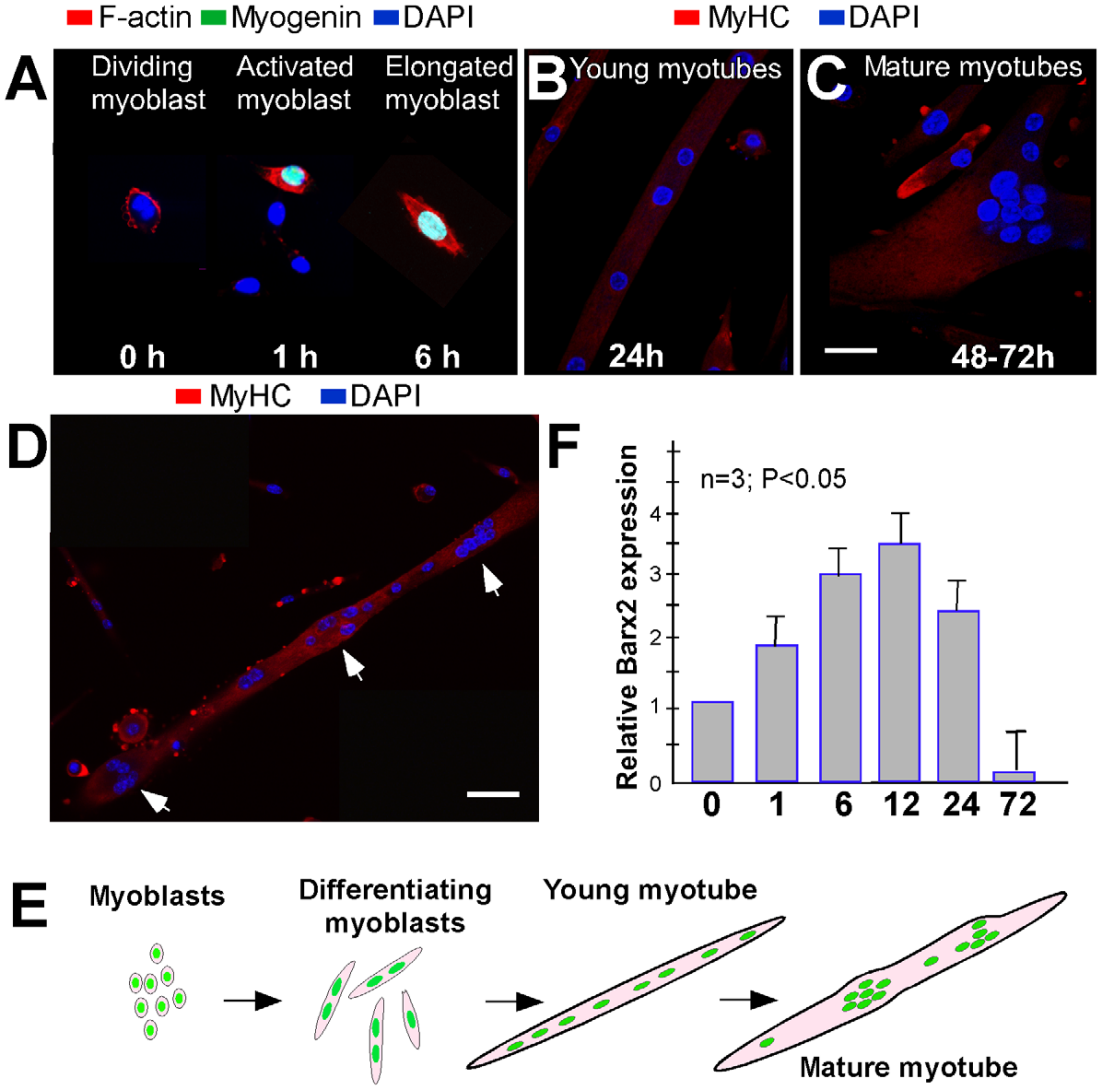
Differentiating myoblasts and maturating myotubes have different levels of Barx2 expression. **A.** Cultured primary myoblasts were induced to differentiate by serum withdrawal and changes in cell shape, actin remodeling and myogenin expression were monitored over the first 6 hours. **B–D.** Myotube maturation was examined between 24 and 72 hours post serum-withdrawal. At 24 hours myotubes were thin and appeared immature (**B**). Between 48 and 72 hours, thicker myotubes appeared often with local aggregations of nuclei (**C** and **D**) and frequent strong contractions. **E.** Observations of myotube maturation in culture presented schematically. **F.** Barx2 expression was measured by RT-PCR at different stages of differentiation. Scale bars represent (A–C) - 20 µm, D–50 µm.

Satellite cells were isolated from postnatal day four mouse skeletal muscle [30], [32] and cultured as proliferating myoblasts. Activation of primary myoblasts by serum withdrawal [30] induced the expected rapid changes in cell shape, remodeling of the actin cytoskeleton and expression of differentiation markers (Fig. 2A). Specifically, within 1–3 hours after serum withdrawal, myogenin expression was induced in activated myoblasts that were undergoing cytoskeletal rearrangement as indicated by phalloidin staining for F-actin [30] (Fig. 2A). We observed that myotube formation/maturation in primary cultures happened in two distinct stages. First long, thin, myotubes were formed. In these myotubes nuclei were aligned toward the middle of the fiber (see Fig. 2B) and the myotubes showed slow, infrequent contractions. We considered these stage 1 or immature (young) fibers. These immature myotubes appeared to be quite stable and did not show any signs of spontaneous disassembly, such as we have observed in forming myotubes by time-lapse microscopy [30]. Later the majority of myotubes thickened and the nuclei moved along the axis of the tube and often formed local aggregations either in the middle or towards the end of the myotube (see Fig. 2C and D). These more mature myotubes contracted more strongly and frequently and appeared to represent the final stage (stage 2) of primary myoblast differentiation. Our observations are presented schematically in Figure 2E. Interestingly, even in very long-term (two week) cultures, thin, slowly contracting myotubes without nuclei clusters persisted in the culture. The existence of two similar types of myotubes with and without nuclei clustering was described previously [33]. Moreover more myotubes with nuclei clusters and larger nuclei aggregates were observed when myotubes were co-cultured with neurons [33]. We also analyzed *Barx2* expression during primary myotube formation; specifically Barx2 mRNA levels were measured by RT-PCR at different times after activation of differentiation by serum withdrawal. Undifferentiated proliferating primary myoblasts expressed Barx2 and its expression was increased at least 3-fold by 6–9 hours after serum withdrawal (Fig. 2F). Subsequently *Barx2* expression levels were reduced several fold below that of undifferentiated myoblasts (Fig. 2F). This is consistent with our previous work [30], [31] indicating that *Barx2* is important early in the myoblast differentiation process and is subsequently downregulated in mature myotubes. In particular, we previously found that ectopic *Barx2* expression in both undifferentiated C2C12 myoblasts and primary cells increased proliferation and accelerated differentiation [30], however it remained unclear why *Barx2* expression is strongly downregulated in myofibers.

**Figure 3:**
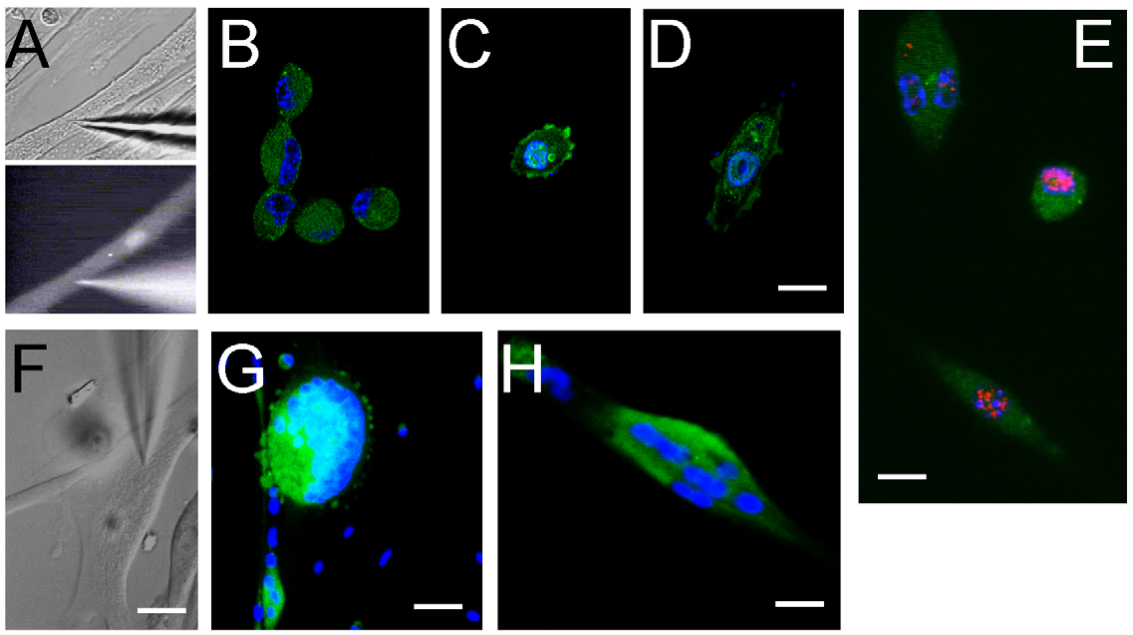
Barx2 induces cleavage of mouse myotubes. **A–D.** Barx2/pcDNA3 or empty pcDNA3 plasmids were mixed with Alexa Fluor®488-conjugated dextran and then injected into immature (stage 1) myotubes. Cultures were stimulated with growth medium and fluorescently labeled myotubes were monitored for signs of cleavage and appearance of labeled mononucleated cells. Scale bar represent 10 µm. **E.** BrdU labeling after microinjection of myotubes. Single Alexa Fluor®488-conjugated dextran-labeled cells occasionally incorporated BrdU suggesting they has re-entered the cell cycle. Scale bar represent 10 µm. **F–H**. Mature (stage 2) myotubes were microinjected and examined as in A-D. No myotube cleavage was observed. Scale bars represent F–10 µm, G - 50 µm, H–20 µm.

To assess whether *Barx2* can regulate plasticity of primary myotubes, we isolated primary mouse myoblasts [32] and induced their differentiation by serum withdrawal as described above. The analysis of these cultures showed that even fully differentiated cultures retain some undifferentiated mononucleated cells even 72–96 hours after induction of differentiation. As with the experiments presented in Figures 1 and 2, this heterogeneity could confound our results, i.e. newly proliferating cells might derive from dedifferentiation of myotubes or from amplification of existing mononucleated cells. To overcome this problem we decided to perform experiments on single myotubes using microinjection of plasmid DNA together with a cell tracing dye (Fig. 3).

We performed injections of Barx2 cDNA or control plasmid (empty pcDNA3 vector) into immature (stage 1) or mature (stage 2) myotubes (Fig. 3A–E, and 3F–H respectively). Injections were performed using a patch-clamp apparatus (see Materials and Methods). To visualize and track injected myotubes, pcDNA3-Barx2 or empty pcDNA3 (control) expression plasmids were mixed with Alexa Fluor® 488-conjugated dextran. To maximize any proliferative response, both sets of injected myotubes were subsequently stimulated with growth medium and medium was replaced daily. Dedifferentiation was assessed by morphologic examination of fluorescently labeled myotubes for signs of cleavage, and by the appearance of myotube-derived (i.e. fluorescently-labeled) mononucleated cells. Cleavage of young myotubes was observed at day 4 after injection of the Barx2 plasmid yielding labeled, mononucleated cells (Figure 3A–D). An example of a young injected multinucleated myotube that cleaved to form five adjacent fluorescently labeled mononucleated cells is shown in Figure 3B. Some of these fluorescently labeled mononucleated cells derived from injected myofiber incorporated BrdU (Fig. 3E). This finding suggests that the mononucleated cells that arise from fragmentation of Barx2-injected myotubes are viable and able to proliferate. Unfortunately the dye did not persist long enough in the cells to assess whether they would be able to re-differentiate if serum was withdrawn again.

In contrast to the results in immature myotubes, injections of Barx2-plasmid into mature myotubes did not induce cleavage (Fig. 3F–H); however, it did tend to induce contraction, nuclei segregation and often myotube death. There was no sign of myotube cleavage or contractions in either immature or mature myotubes injected with empty plasmid DNA (not shown). Overall these data indicate that *Barx2* can promote dedifferentiation of single immature myotubes.

**Figure 4:**
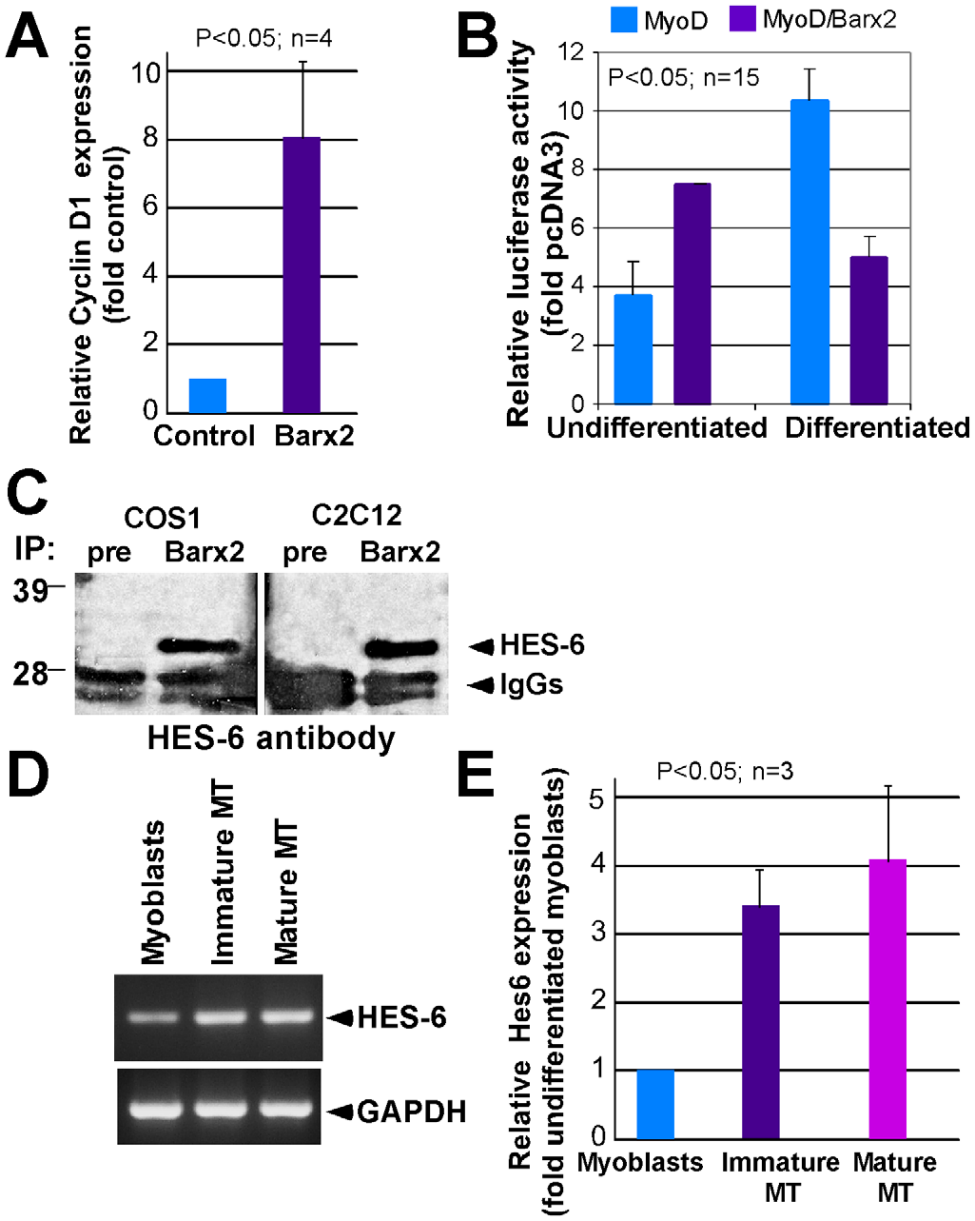
Regulatory connections of Barx2 during muscle differentiation. **A.** Quantitative RT-PCR analysis of cyclin D1 expression in C2C12 cells stably transfected with a Barx2-expression plasmid or control plasmid. **B.** The SMA promoter-luciferase construct was co-transfected with combinations of Barx2, MyoD, or empty pcDNA3 expression plasmids in C2C12 cells. Barx2 increased SMA promoter activation in undifferentiated cell cultures yet inhibited activation in myotubes. **C.** Co-immunoprecipitation was performed after expression of both Barx2 and Hes6 proteins in COS1 cells and C2C12 cells. Barx2 co-immunoprecipitated Hes6 in both contexts. **D.** Expression of Hes6 was compared in C2C12 myoblasts and myotubes using RT-PCR. Hes6 is upregulated in myotubes. **E**. Graphical representation of the RT PCR data shown in **D** (n – number of experiments).

Because our BrdU incorporation data suggested that *Barx2* could induce re-entry of myotube-derived cells into the cell cycle, we examined whether *Barx2* might affect cell cycle genes directly. Quantitative RT-PCR analysis of C2C12 cells stably transfected with a Barx2-expression plasmid showed that the cyclin D1 gene was upregulated approximately 9-fold relative to control-transfected cells (Fig. 4A). The D-type cyclins drive cells through the G0-G1-S checkpoint, thus allowing quiescent cells to reenter the cell cycle [34], which would be consistent with a role for reactivating previously quiescent myonuclei after myotube dedifferentiation.

The data presented here combined with our previous work [30], [31] suggest that ectopic expression of *Barx2* can have different effects at different stages of myoblast differentiation. At an early stage of myoblast differentiation, increased expression of Barx2 upregulates the expression of several myoblast differentiation markers including α-smooth muscle actin (SMA) and accelerates differentiation [30], [31], while in myotubes, ectopic expression of Barx2 appears to suppress the expression of differentiation-associated genes (Fig. 1, 3). To further explore this notion of differential gene regulation, we compared the regulation of a SMA promoter-luciferase reporter construct by Barx2 in undifferentiated myoblasts or differentiated myotubes (Fig. 4B). Endogenous *SMA* expression is known to be upregulated at an early stage of myoblast differentiation, possibly to facilitate migration and remodeling, and is subsequently downregulated and replaced by skeletal muscle actin [35]. Moreover, we have previously shown that Barx2 regulates the endogenous *SMA* gene and can directly bind to the SMA promoter together with MyoD and increase transcriptional activation of the promoter by MyoD in undifferentiated myoblasts [30].

The regulation of the *SMA* promoter by Barx2 and MyoD was examined by co-transfection of the SMA promoter-luciferase construct with Barx2, MyoD, or empty pcDNA3 expression plasmids in C2C12 cells (Fig. 4B). Consistent with our previous studies [30], Barx2 increased activation of the *SMA* promoter by MyoD in undifferentiated cell cultures. However, when promoter activity was assayed after differentiation into myotubes, Barx2 was found to moderately inhibit activation of the *SMA* promoter by MyoD (Fig. 4B). This suggests that *Barx2* may promote muscle gene expression in myoblasts, yet repress the same and/or other genes after differentiation.

We previously showed that Barx2 contains both an N-terminal repression domain and a C-terminal activation domain [36] that may recruit various co-repressors and co-activators respectively. We also found that Barx2 interacts directly with positive regulators of myogenesis including the bHLH factor MyoD, and its coactivators including CREB-binding protein (CBP) [30]. Our new observation that *Barx2* can both up and downregulate the *SMA* promoter depending on the state of cellular differentiation prompted us to examine whether it may also interact with negative bHLH regulatory factors.

The Hairy-enhancer of split (HES) family of bHLH factors act as transcriptional repressors and have roles in maintaining progenitor cells in an undifferentiated state and regulating cell fate decisions during embryogenesis. For example, lack of certain *HES* genes leads to premature differentiation in the nervous system [37], [38], [39]. *Hes6* has been previously shown to be an inhibitor of myoblast differentiation in C2C12 myoblasts [40]. To assess whether Barx2 and Hes6 can interact, co-immunoprecipitation was performed after expression of both Barx2 and Hes6 proteins in COS1 cells and C2C12 cells. We found that Barx2 can co-immunoprecipitate Hes6 in both contexts (Fig. 4C). Taken together with our previous work [30], we conclude that Barx2 can form complexes with both positive (i.e. MyoD) and negative (i.e, Hes6) bHLH myogenic regulators. We also examined the relative expression of Hes6 in C2C12 myoblasts and myotubes using RT-PCR and found that Hes6 expression was relatively low in undifferentiated C2C12 cells and was moderately upregulated in both immature and mature myotubes (Fig. 4D). This result is consistent with a previous non-quantitative analysis of Hes6 expression in C2C12 cells showing increased Hes6 mRNA after induction of differentiation [40]. Our data prompt the hypothesis that differential expression of factors such as *Hes6*, or other Barx2-interacting repressors yet to be identified, could contribute to the dual function of *Barx2* at different stages of muscle development.

## Discussion

The homeobox protein Barx2 is expressed during development of skeletal and smooth muscle [41], [42]. Barx2 is expressed in primary myoblasts and its expression is upregulated soon after induction of differentiation, while differentiated myotubes express virtually no Barx2. This not only suggested a role for *Barx2* early in myoblast differentiation as previously investigated [30], [31], but also that subsequent repression of endogenous Barx2 in myofibres might be important for their function and/or integrity. Here we found that ectopic expression of Barx2 in differentiated myotubes originating from either cell lines or isolated muscle progenitors induced myotube cleavage and downregulated differentiation markers. We were also able to demonstrate increased cell proliferation after ectopic Barx2 expression and using microinjection and cell tracing show that proliferative cells arose from dedifferentiated myotubes.

These finding were similar to the previously described functions of another homeodomain transcription factor - *Msx1* [9], [25]. Moreover, the regenerative plasticity of isolated urodele myofibers is dependent on *Msx1* [9] and *Msx1* is involved in epimorphic digit tip regeneration in both humans and mice [21], [23]. It is possible that *Barx2* and *Msx1* share similar pathways and even target genes in the regulation of dedifferentiation. However, an important difference between the functions of *Barx2* and *Msx1* is that *Barx2* is also able to promote the early stages of myoblast differentiation in cooperation with *MyoD* [30]. This has not been reported for *Msx1*; on the contrary expression of *Msx1* in undifferentiated C2C12 myoblasts downregulates *MyoD* expression and inhibits differentiation into myotubes [22], [25], [43]. Ectopic expression of Msx1 in the forelimb and somites of chicken embryos also inhibits MyoD expression and muscle differentiation [22].

It is interesting that in addition to *Msx1* a number of homeobox factors are capable of suppressing myogenic differentiation. These include *Mohawk* which is a transcriptional repressor that blocks the myogenic conversion of C3H10T1/2 cells [44]. The *Pax3* and *Pax7* paired homeodomain transcription factors have also been shown to suppress myogenic differentiation and both the paired domain and homeodomain of *Pax3* are required for this anti-myogenic effect [45]. Thus, the *Msx1, Mohawk*, and *Pax3/7* homeobox containing transcription factors appear to be general repressors of myoblast differentiation. In contrast our previous and current studies indicate that *Barx2* positively regulates the early steps of myoblast differentiation; however, its subsequent down-regulation may be necessary for maintenance of the differentiated state of myofibers.

The upregulation of cyclin D1 expression after overexpression of *Barx2* in C2C12 cells could be an important factor in promoting cell cycle re-entry by myonuclei derived from dedifferentiated myofibres. Re-entry into the cell cycle has been also induced in terminally differentiated cultured cardiomyocytes by expression of G1 cell cycle factors [46]. It is also of note that the highly regenerative ‘scarless’ MRL mouse, which shares some properties with newts and zebrafish in the formation of wound blastema and apparent regeneration of heart muscle, was recently shown to lack expression of p21 in regenerating tissue [47]. Moreover, p21 null mice were recently shown to have a highly regenerative phenotype similar to that of MRL mice. p21 is an inhibitor of cdk2, which is involved in G1-S transition [48], supporting the idea that modulation of genes that control cell cycle checkpoints might alter the program of differentiated cells and allow increased plasticity. Future work will examine how *Barx2* regulates a transcriptional program that allows cellular remodeling to be coordinated with cell cycle re-entry.

Although mechanisms underlying differential gene regulation by *Barx2* at early and late stages of muscle differentiation are unclear, they are very likely to involve differential recruitment of activator or repressor complexes by Barx2 [36]. As we showed in previous [30], [36] and current studies, Barx2 can form complexes with both positive and negative bHLH regulators of myogenesis; i.e. MyoD and Hes6. The ratios between these complexes may thus be important for determining *Barx2* function at different stages of myogenic differentiation.

Enforced expression of Hes6 has been found to inhibit differentiation, leading to a higher proportion of thin, immature myotubes relative to thickened, mature myotubes. Hes6 expression also reduced the proportion of cells undergoing cell cycle withdrawal and allowing more cells to re-enter the cell cycle after differentiation [40]. These activities were independent of the DNA binding domain of Hes6 and most likely depend on protein-protein interactions [40]. Moreover, when injected into *Xenopus* embryos, Hes6 increased the size of the myotome due to increased proliferation; it also decreased markers of terminal muscle differentiation [40]. While upregulated early in differentiation, Hes6 is reported to be absent in mature myofibers in vivo, leading to the suggestion that downregulation of Hes6 is required for acquisition of a stable terminal differentiated state [40]. This closely parallels the situation that we observe with Barx2.

Our corroborating finding of increased *Hes6* expression in C2C12 myotubes relative to myoblasts [40], together with the interaction of Barx2 and Hes6, suggests that Hes6 may be one of the repressors that interact with ectopic Barx2 in immature myotubes and helps suppress muscle specific gene expression. This interaction could contribute to suppression of myotube maturation and allow immature myotubes to undergo dedifferentiation. Consistent with the idea of functional cooperation between *Barx2* and *Hes6,* previous work showed that the repression domain of Barx2 binds directly to the transducin-like enhancer of split (TLE) corepressor family [49]. TLEs are also essential corepressors for *Hes6* and other HES family repressors [49]. The role of Barx2-Hes-TLE interactions in repression of gene expression is under further investigation. Moreover, additional mechanisms for repression of myogenic gene expression and reversal of the differentiated state are also likely.

Homeodomain transcription factors regulate gene expression in response to a large variety of extracellular stimuli, and act as molecular switches for controlling cell differentiation, proliferation, and apoptosis. Particular homeobox genes (*Oct, Nanog*) have been shown to be important in induction of pluripotent stem cells (iPS cells). Recent work comparing *zebrafish* regeneration blastema with iPS cells showed that, although blastemal cells are not pluripotent, some of the key iPS reprogramming factors including the Pou5 homeobox protein are also important for regeneration, presumably due to their role in generating a multipotent cell state. This suggests some common mechanisms may be involved in induced cellular reprogramming and in the natural dedifferentiation process observed in the blastema [50]. We now add *Barx2* to the list of homeobox regulators of adult cellular plasticity. In future work it would be of considerable interest to assess the role of *Barx2* in blastemal regeneration of muscle, perhaps in the *zebrafish* context.

## Materials and Methods

### Cell cultures

Mouse C3H10T1/2 (clone 8) and C2C12 (ATCC) cells were grown in Dulbecco's Modified Eagle's Medium (DMEM) supplemented with 10% heat-inactivated fetal bovine serum (FBS) at 37C in a humidified 5% CO2 atmosphere without antibiotics.

### Preparation of primary myoblasts and immunostaining

Primary myoblast cultures were prepared as described previously [32]. Cells were grown on collagen-coated plates or chamber slides and maintained in growth medium (1∶1 Ham's F10/DMEM, supplemented with 20% FBS and 2.5 ng/ml of basic FGF). Cells were differentiated by transfer into differentiation medium (DMEM supplemented with 2% horse serum). Cells were fixed with 2% of paraformaldehyde at various time points after induction of differentiation and processed for immunostaining. Antibodies to myogenin (clone F5D; BD Bioscience Pharmingen), α-smooth muscle actin (SMA) (clone 1A4; Sigma) or fast myosin heavy chain (clone MF20 Developmental Studies Hybridoma Bank), were used for immunostaining. Secondary antibodies (Molecular Probes, Invitrogen) were conjugated to Alexa-488 or Texas Red. Rhodamine-conjugated phalloidin was used to visualize F-actin.

### Microscopy and image analysis

The Zeiss LSM 710 laser scanning confocal microscope (LSCM) was used to obtain images. IMARIS software was used for image analysis.

### Cell transfections and promoter assays

1×10^7^ C2C12 cells were transfected with either 10 µg of Barx2/pcDNA3 expression vector or pcDNA3 control plasmid using Lipofectamine2000 reagent (Invitrogen). The Barx2 expression plasmid contains an in-frame NH_2_-terminal Myc tag and was described previously [41]. In other experiments, an expression plasmid bearing mCherry fluorescent protein linked to Barx2 via a ‘self-cleaving’ 2A peptide sequence was used to generate stable lines by selection with puromycin in C2C12 cells. C3H10T1/2 cells were co-transfected with MyoD and Barx2-pcDNA3 or with MyoD and pcDNA3 plasmids. For analysis of smooth muscle actin (SMA) promoter activity, a SMA promoter-luciferase construct [30] was co-transfected with the Barx2-pcDNA3 expression vector or pcDNA3 control plasmid and harvested either before or after differentiation. The data shown were derived from at least three independent experiments. Luciferase activity was analyzed as previously described [30].

### Western blotting

Total protein was prepared from C2C12 and C3H10T1/2 cells using RIPA lysis buffer and sonicated. Equal aliquots of protein were resolved by SDS-PAGE, transferred to polyvinylidene difluoride membrane, and probed with antibodies to MyoD (clone MoAb5.8A; BD PharMingen), skeletal fast myosin (clone MY-32; Sigma), myogenin (clone F5D; BD Bioscience Pharmingen) and a custom-made Barx2 anti-peptide polyclonal antibody (Covance). β-actin, antibody was used as a reference. HRP conjugated secondary antibodies and a chemiluminescent detection system was used to visualize proteins. All experiments were performed in duplicates.

### Co-Immunoprecipitation

Co-immunoprecipitation of Barx2 with Hes6 was performed as essentially as described in [Zorn, 1999] Briefly, COS1 or C2C12 cells were co-transfected with the Barx2/pcDNA3 and Hes6/pCMVSport6 (Open Biosystems) expression plasmids using Lipofectamine-2000 (Invitrogen). Cell lysates were prepared 48 hours after transfection, pre-cleared with Protein A-Sepharose and then incubated overnight at 4°C with 5 µg of anti-Barx2 rabbit antibody or preimmune serum. Complexes were precipitated with Protein A-Sepharose, washed four times and resolved by SDS-PAGE and immunoblotted with anti-Hes6 polyclonal goat antibody (Santa Cruz Biotechnologies) antibody.

### RNA isolation and RT PCR

RNA was isolated from undifferentiated C2C12 cells and C2C12 young and mature myotubes using the RNeasy Plus Mini Kit (Qiagen). The RNA was quantified using a Beckman DU 640 Spectrophotometer. 1 µg of each RNA sample was used to synthesize cDNA using a First-Strand cDNA Synthesis kit and SuperScript III/RNaseOUT Enzyme mix and 50 ng/ul random hexamer primers. The RT PCR was performed using Perkin Elmer 9600 PCR machine. Each sample was amplified with Hes6 primers: forward: ctcctgaaccacctgctagaatcc, reverse: ctaaggatgtagacaccaaatccggc, and GAPDH primers: forward: gtgaaggtcggtgtgaacggatttggccg; reverse: ccatggtggtgaagacaccagtagactcc. The Hes6 primer set amplified a 252 base-pair DNA segment of the Hes6 cDNA. The PCR products were resolved by agarose electrophoresis, bands were quantified by densitometry, and the ratio of Hes6/GAPDH PCR products was calculated. Three experiments were performed, and the results were normalized to the values from undifferentiated myoblast cultures. Statistical (t-test) analysis was performed using Microsoft Exel.

### Quantitative RT-PCR

RNA was prepared from primary myoblast or C2C12 cell cultures using Trizol reagent (Gibco). Cells were lysed directly in Trizol and RNA was prepared according to manufacturer protocol (Invitrogen). RNA was treated with DNase using the DNA-free kit (Ambion) and reverse transcribed using random primers and MMluV reverse transcriptase (New England Biolabs). Quantitative RT-PCR reactions were performed on an ABI 7300 machine using Superarray Biosciences RT2 SYBR green reagent and the following primers. Mouse Barx2 F: gtatttgtctaccccagacaggtt, R: tcatcctgcgattctgatacc; mouse Cyclin D1 F: tctttccagagtcatcaagtgtg, R: gactccagaagggcttcaatc. Mouse GAPDH (NM_008084) (QIAGEN SaBiosciences) and mouse ribosomal protein S26 (RPS26) F: aggtgcagaaggctgagg, R: ggttctcccgagtgatgaag, were used as controls.

### Intracellular injections

Injections into myofibers were performed using traditional patch-clamp electrophysiology equipment [51]. Glass micropipettes (1 mm outer diameter/0.58 internal diameter, WPI Inc., Sarasota, FL) were pulled on a Sutter P97 Flaming/Brown puller (Sutter Instruments, Novato, CA). Tips were approximately 1 mm in diameter, and had a resistance in the bath of 2–5 MW. Micropipettes were backfilled with internal patch solution containing (in mM) K-gluconate 110, KCl 10, HEPES 10, Phosphocreatine 10, Mg-ATP 4, Na-GTP 0.3, Biocytin 0.1%, pH 7.3, with and osmolarity of 280–290 mOsm. The Barx2-pcDNA3 and pcDNA (control) plasmids were mixed with fixable Alexa Fluor® 488-conjugated dextran; 10,000 kDa; (Invitrogen) and added at a concentration of 0.5 µg/ml. Bath solution contained (in mM) NaCl 124, KCl 3, NaPO_4_ 1.25, NaHCO_3_ 26, MgCl_2_ 1, Glucose 25, CaCl_2_ 2.

### BrdU labeling experiments

In experiments to assess the proliferation of cells derived from microinjected myotubes, the cultures were maintained for an additional 4–5 days and then incubated with 5-bromo-2-deoxyuridine (BrdU) (Cell Proliferation Kit RPN20; Amersham Biosciences) for one hour. Cultures were then fixed and BrdU detection was performed using an Alexa Fluor® 594-conjugated secondary antibody according to the manufacturers protocol. Proliferating BrdU-labeled cells were detected as fluorescein-positive cells by confocal microscopy.
